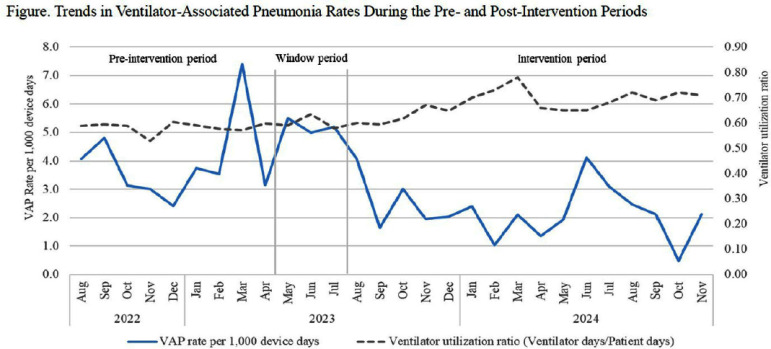# Evaluation of head elevation and oral care enhancement activity in intensive care units to prevent ventilator-associated pneumonia

**DOI:** 10.1017/ash.2025.209

**Published:** 2025-09-24

**Authors:** Seung Hee Ryu, Eunbyeol Jo, Soyeon Park, Miseo Kim, Ja Young Kim, Hyunju Lee, Subeen Moon, Jeeyoon Kim, Jiwon Jung

**Affiliations:** 1 Asan medical center

## Abstract

**Background:** Ventilator-associated pneumonia (VAP) primarily occurs due to the aspiration of secretions containing microorganisms from the oropharynx or stomach into the lungs. Preventing aspiration is a critical strategy for reducing VAP incidence. This study analyzed the impact of aspiration prevention measures—head-of-bed elevation (HOBE) and enhanced oral care—on VAP rates in adult intensive care units (ICUs). **Method:** This interventional study was conducted in the adult ICU of a 2,734-bed tertiary care hospital. A total of 8 ICUs (medical, surgical, cardiology, cardiovascular, neurology and neurosurgery) with 112 beds observed an increase in VAP incidence from January to April 2023, prompting enhanced measures in May 2023. The first intervention involved revising and reinforcing indications for head-of-bed elevation (HOBE) while strengthening monitoring and on-site feedback. During clinical procedures such as positional changes requiring a supine position, oropharyngeal suctioning was performed before lowering the head of the bed, and staff were trained to ensure prompt restoration of the HOBE to the appropriate position afterward. The second intervention improved oral care by replacing chlorhexidine and gauze with tooth brushing. A protocol was developed requiring 2 minutes of brushing teeth, artificial airways, tongue, and palate using a silicone toothbrush moistened with saline or sterile water, excluding patients with contraindications such as bleeding risks. Monitoring revealed missed areas during brushing, necessitating additional simulation training using dental models and colored toothpaste to confirm plaque removal. The pre-intervention period was conducted over 9 months (August 2022 to April 2023), while the intervention period lasted 17 months (July 2023 to November 2024). VAP incidence rates were compared before and after the intervention. Additionally, the incidence of VAP associated with pathogens such as Klebsiella pneumoniae, Acinetobacter baumannii, or Pseudomonas aeruginosa, often isolated from dental plaques of ICU patients, were analyzed. **Results:** The incidence rate of VAP per 1,000 ventilator days among adult ICU patients decreased from 3.9 (66/16,849) before the intervention to 2.4 (78/32,185) after the intervention (IRR, 0.62, 95% CI, 0.45-0.86; P = 0.007). Similarly, the incidence rate of VAP associated with pathogens K. pneumoniae, A. baumannii, or P. aeruginosa were 1.6 (27/16,849) before the intervention, and 1.0 (31/32,185) after the intervention (IRR, 0.60, 95% CI, 0.36-1.01; P = 0.07). **Conclusion:** As a result of implementing enhanced head-of-bed elevation and oral care protocols for ventilated patients in the adult ICU, the incidence of VAP significantly decreased. Further multicenter studies are needed to validate our findings.

**Figure:**